# Virologic Failure and Drug Resistance After Programmatic Switching to Dolutegravir-based First-line Antiretroviral Therapy in Malawi and Zambia

**DOI:** 10.1093/cid/ciae261

**Published:** 2024-06-07

**Authors:** Veronika Whitesell Skrivankova, Jacqueline Huwa, Guy Muula, Geldert D Chiwaya, Esau Banda, Shameem Buleya, Belinda Chihota, Joseph Chintedza, Carolyn Bolton, Hannock Tweya, Thokozani Kalua, Stefanie Hossmann, Roger Kouyos, Gilles Wandeler, Matthias Egger, Richard J Lessells

**Affiliations:** Institute of Social and Preventive Medicine, University of Bern, Bern, Switzerland; Lighthouse Trust, Lilongwe, Malawi; Centre for Infectious Disease Research in Zambia, Lusaka, Zambia; Lighthouse Trust, Lilongwe, Malawi; Centre for Infectious Disease Research in Zambia, Lusaka, Zambia; Lighthouse Trust, Lilongwe, Malawi; Centre for Infectious Disease Research in Zambia, Lusaka, Zambia; Lighthouse Trust, Lilongwe, Malawi; Centre for Infectious Disease Research in Zambia, Lusaka, Zambia; Lighthouse Trust, Lilongwe, Malawi; International Training and Education Center for Health (I-TECH), Lilongwe, Malawi; Center for International Health, Education, and Biosecurity (Ciheb) at University of Maryland, Baltimore School of Medicine (UMB), Lilongwe, Malawi; Institute of Social and Preventive Medicine, University of Bern, Bern, Switzerland; Diabetes Center Berne, Bern, Switzerland; Department of Infectious Diseases and Hospital Epidemiology, University Hospital Zurich, University of Zurich, Zurich, Switzerland; Institute of Medical Virology, University of Zurich, Zurich, Switzerland; Institute of Social and Preventive Medicine, University of Bern, Bern, Switzerland; Department of Infectious Diseases, Bern University Hospital, University of Bern, Bern, Switzerland; Institute of Social and Preventive Medicine, University of Bern, Bern, Switzerland; Population Health Sciences, Bristol Medical School, University of Bristol, Bristol, United Kingdom; Centre for Infectious Disease Epidemiology and Research, Faculty of Health Sciences, University of Cape Town, Cape Town, South Africa; KwaZulu-Natal Research Innovation and Sequencing Platform (KRISP), School of Laboratory Medicine & Medical Sciences, University of KwaZulu-Natal, Durban, South Africa; Centre for the AIDS Programme of Research in South Africa (CAPRISA), University of KwaZulu-Natal, Durban, South Africa

**Keywords:** HIV, antiretroviral therapy, dolutegravir, drug resistance, Southern Africa

## Abstract

**Background:**

People with human immunodeficiency virus (PWH) on first-line, nonnucleoside reverse-transcriptase inhibitor–based antiretroviral therapy (ART) were routinely switched to tenofovir-lamivudine-dolutegravir. We examined virologic outcomes and drug resistance in ART programs in Malawi, where switching was irrespective of viral load, and Zambia, where switching depended on a viral load <1000 copies/mL in the past year.

**Methods:**

We compared the risk of viremia (≥400 copies/mL) at 1 and 2 years by viral load at switch and between countries using exact methods and logistic regression adjusted for age and sex. We performed HIV-1 *pol* Sanger sequencing on plasma samples with viral load ≥1000 copies/mL.

**Results:**

A total of 2832 PWH were eligible (Malawi 1422, Zambia 1410); the median age was 37 years, and 2578 (91.0%) were women. At switch, 77 (5.4%) were viremic in Malawi and 42 (3.0%) in Zambia (*P* = .001). Viremia at switch was associated with viremia at 1 year (adjusted odds ratio (OR), 6.15; 95% confidence interval [CI], 3.13–11.4) and 2 years (7.0; 95% CI, 3.73–12.6). Viremia was less likely in Zambia than in Malawi at 1 year (OR, 0.55; 0.32–0.94) and 2 years (OR, 0.33; 0.18–0.57). Integrase sequencing was successful for 79 of 113 eligible samples. Drug resistance mutations were found in 5 PWH (Malawi 4, Zambia 1); 2 had major mutations (G118R, E138K, T66A and G118R, E138K) leading to high-level dolutegravir resistance.

**Conclusions:**

Restricting switching to dolutegravir-based ART to PWH with a viral load <1000 copies/mL may reduce subsequent viremia and, consequently, the emergence of dolutegravir drug resistance mutations.

**Clinical Trials Registration:**

Clinicaltrials.gov (NCT04612452).

## Teaser

We compared programmatic switching to dolutegravir-based first-line antiretroviral therapy under different guidelines. The risk of subsequent viremia was higher in those switching with viremia at switch. It was reduced where switching was restricted to those with recently suppressed viral load.

Since 2018, the World Health Organization (WHO) has recommended the integrase strand transfer inhibitor (INSTI) dolutegravir (DTG) as the third drug in first-line antiretroviral therapy (ART) for people with human immunodeficiency virus (HIV) (PWH), owing to its effectiveness in suppressing viral replication and its high genetic barrier to resistance [1–3]. DTG in first-line ART is more successful in suppressing viral replication than the use of efavirenz-based regimens [4–6]. The DAWNING [7] and nucleosides and darunavir/dolutegravir in Africa (NADIA) [8] trials showed that when combined with at least 1 fully active nucleoside reverse transcriptase inhibitor (NRTI), DTG-based ART is effective in second-line therapy. The use of DTG should bring down the cost of first-line ART and reduce the demand for expensive second- and third-line regimens [9, 10].

The WHO guidelines advise viral load (VL) testing for patients on ART at least once per year. They also recommend that VL should be <1000 copies/mL before transitioning from nonnucleoside reverse transcriptase inhibitor (NNRTI)-based first-line ART to DTG-based ART [2, 11]. Most PWH with virologic failure on first-line NNRTI-based ART have NRTI mutations, which in turn may increase the risk of DTG resistance [12, 13]. DTG combined with tenofovir disoproxil fumarate and lamivudine (TLD) has been rolled out as a first-line regimen in most countries in sub-Saharan Africa. However, PWH are often switched without confirmation of virological suppression, and genotypic drug resistance testing at switch is rarely performed [14, 15]. This programmatic approach in resource-limited settings may thus have led to HIV-1 viremic PWH being switched to DTG-based ART [13, 16].

We report the results from the DTG SWITCH study (Clinicaltrials.gov NCT04612452), which examined viremia and antiretroviral resistance among adult PWH programmatically switched to DTG-based first-line ART in 2 countries: Malawi, where PWH were switched regardless of their VL and Zambia, where only PWH whose last VL was <1000 copies/mL were transferred to DTG-based ART.

## METHODS

DTG SWITCH studied PWH on first-line, NNRTI-based ART who switched to dolutegravir-based ART in 2019 to 2021 in Malawi or Zambia, with assessments of viremia and dolutegravir resistance at 1 year and 2 years.

### Study Sites

The study recruited patients from 2 large ART programs of the International epidemiology Databases to Evaluate AIDS (IeDEA) in Southern Africa [17]. The Lighthouse Trust [18] is a public trust based in Lilongwe, Malawi, that contributes to Malawi's national response to HIV as a partner of the government's HIV program. It operates 5 centers (1 each in Blantyre, Zomba, Mzuzu, and 2 in Lilongwe) for integrated HIV testing, treatment, and care. The two Lighthouse clinics from Lilongwe participated in DTG SWITCH. The Centre for Infectious Disease Research in Zambia (CIDRZ) [19] is a nongovernmental health organization based in Lusaka, Zambia, that has been an active partner of the Government of the Republic of Zambia through the Ministry of Health since 2001. Its primary focus is on infectious diseases, particularly HIV/acquired immunodeficiency syndrome (AIDS), malaria, and tuberculosis. Three CIDRZ clinics from Lusaka participated in DTG SWITCH.

### National Guidelines

In both countries, national guidelines recommended that adult PWH on first-line ART are switched to TLD, except for women of childbearing age. In 2019, the recommendation to switch was extended to women younger than age 45 years. In Malawi, eligible PWH were generally switched regardless of their VL value [20]. In Zambia, the national guidelines recommended only switching patients who had a VL value <1000 copies/mL [21] within the past year.

### Patient Eligibility

PWH were eligible if they were aged 18 years or older, had been on an efavirenz- or nevirapine-based first-line ART for at least 6 months, and were switching to a DTG-based first-line triple ART regimen, typically TLD. Patients were recruited to the study consecutively.

### Data Collection

Study nurses oversaw recruitment and data collection. At switch to DTG and at 1 year and 2 years after switching, study participants provided a blood sample, which was tested for VL and for drug resistance at 1 and 2 years if VL was >1000 copies/mL. Study samples were collected in addition to routine care; results were shared with the clinics after the conclusion of the study. Clinical, demographic, and laboratory data were collected using REDCap electronic data capture [22].

### Laboratory Methods

Viral loads were determined at local laboratories using nucleic-acid amplification-based tests. Plasma samples were stored at −70 °C or −80 °C and shipped in batches to the KwaZulu-Natal Research Innovation and Sequencing Platform at the University of KwaZulu-Natal, South Africa [23, 24]. HIV-1 *pol* Sanger sequencing was done on plasma samples using the Applied Biosystems TaqPath Seq HIV-1 Genotyping Kit (Thermo Fisher Scientific, Carlsbad, CA, USA), following the manufacturer's guidelines. Briefly, viral RNA was isolated from 200 µL of concentrated plasma, and the protease/reverse transcriptase and integrase genes were amplified and then sequenced using a 3730xl DNA Analyzer (Thermo Fisher Scientific). Sequences were analyzed for HIV drug-resistance mutations using the Stanford HIV drug resistance database (HIVdb) genotypic resistance interpretation system v9.4 [25, 26].

### Outcomes

Outcomes included HIV-1 viremia defined as VL ≥400 copies/mL and drug resistance mutations. We included major and accessory mutations associated with INSTIs by the Stanford HIVdb algorithm [25, 26]. We used the same approach to assess resistance to all other antiretroviral drugs.

### Statistical Analysis

We used descriptive statistics to compare the characteristics at switch of the 2 study populations. We used chi-squared tests for differences in the prevalence of viremia (VL <400 and ≥ 400 copies/mL) between countries and a Wilcoxon rank-sum test for difference in VL. We visualized VL trajectories from baseline to year 2 and calculated the proportion of PWH with viremia at 1 year and 2 years after switching (within a window of ±90 days) by viremia category at switch. We calculated viremia risk ratios with exact 95% confidence intervals (CI) and *P* values based on binomial distributions of events [27]. We performed logistic regression analyses with viremia at 1 and 2 years as outcomes. Models were adjusted for baseline age (per 10-year increase), sex, baseline VL (<400 vs ≥400 copies/mL), and country. All analyses were performed in R, version 4.1.1 [28].

To test the robustness of the main results, we repeated analyses of viremia with categories <1000 copies/mL vs ≥1000 copies/mL (model S1) and by treating viral load at switch as a continuous variable, modeled as log10-transformed VL with a setoff 10 for undetectable VL measurements (model S2). In the latter model, we additionally included an interaction term between viral load at switch and the country of the ART program (model S3).

The institutional review board of CIDRZ, the Malawi National Health Sciences Research Committee, and the Cantonal Ethics Committee of the Canton of Bern approved this study. All patients provided written informed consent.

## RESULTS

### Characteristics of Study Population

**Figure 1.**
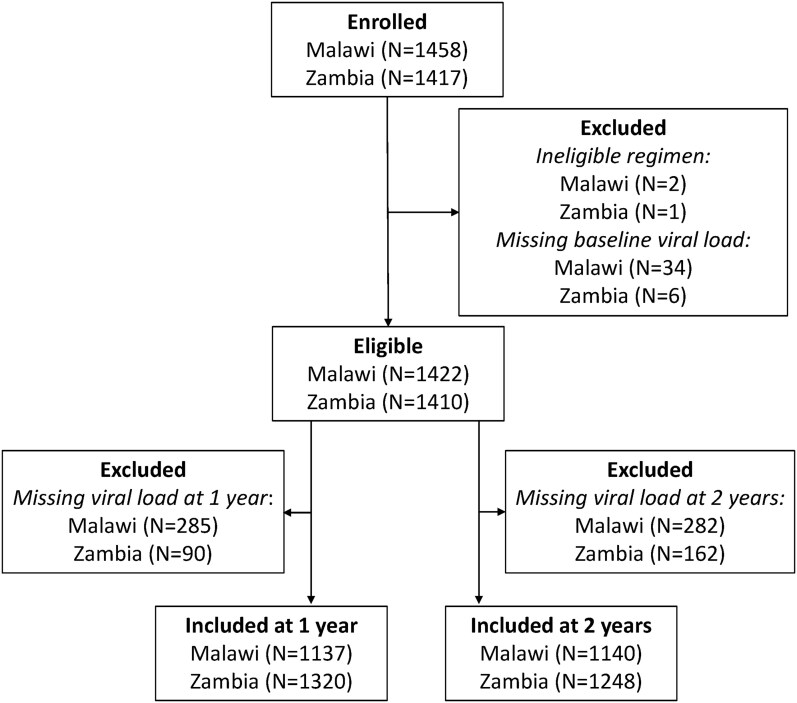
Flow of inclusion of participants into the study.

Of PWH switching during the study period, 1422 of 1458 (97.5%) in Malawi and 1410 of 1417 (99.5%) in Zambia had a VL measurement at switch and were eligible for the analyses (Figure 1). In both countries, most participants were women (Table 1). The median age was lower in Malawi (35 years) than in Zambia (39 years), but the median time on ART was similar in the 2 groups (6 years). The body mass index was around 23 kg/m^2^ in both groups. In Malawi, 90% of PWH were in WHO stage I, compared to 100% in Zambia. Most participants were on ART consisting of efavirenz, lamivudine or emtricitabine, and tenofovir disoproxil fumarate and switched to DTG combined with lamivudine or emtricitabine and tenofovir disoproxil fumarate (Table 1).

**Table 1.** Baseline Characteristics of Study Participants at 2 ART Programs in Malawi and Zambia

	Malawi	Zambia
	N (%) or Median (IQR)	N (%) or Median (IQR)
Total N	1422	1410
Sex		
Women	1409 (99%)	1169 (83%)
Men	13 (1%)	241 (17%)
Age (y)	35 (30–40)	39 (33–45)
Body mass index (kg/m^2^)	23.3 (21.0–26.9)	23.4 (20.8–27.2)
* *Missing	6 (0.4%)	0
WHO stage		
I	1221 (90%)	1408 (100%)
II	64 (4.7%)	0
III	66 (4.9%)	0
IV	4 (0.3%)	0
* *Missing	67 (4.7%)	2 (0.1%)
VL (copies/mL)		
VL < 400	1345 (94.6%)	1368 (97%)
VL ≥ 400	77 (5.4%)	42 (3%)
VL ≥ 1000	64 (4.5%)	26 (1.8%)
Median^a^ (IQR)	8952 (1294–52 750)	1163 (606–11 331)
ART		
Time on ART (y)	6.1 (3.8–9.0)	6.0 (3.5–8.8)
* *Missing	2 (0.1%)	3 (0.2%)
ART regimen before switch		
TDF + XCT + EFV	1385 (97.5%)	1404 (99.6%)
TDF + XTC + NVP	9 (0.6%)	2 (0.1%)
ABC + 3TC + EFV	0	2 (0.1%)
ABC + 3TC + NVP	1 (0.1%)	1 (0.1%)
AZT/d4T + 3TC + EFV	8 (0.6%)	0
AZT/d4T + 3TC + NVP	18 (1.3%)	1 (0.1%)
* *Missing	1 (0.1%)	0
ART regimen after switch		
TDF + XTC + DTG	1419 (99.6%)	1209 (85.8%)
TAF + XTC + DTG	0	201 (14.2%)
ABC + 3TC + DTG	3 (0.2%)	0

Abbreviations: 3TC, lamivudine; ABC, abacavir; ART, antiretroviral therapy; AZT, azidothymidine; DTG, dolutegravir; d4T, stavudine; EFV, efavirenz; FTC, emtricitabine; NVP, nevirapine; TAF, tenofovir alafenamide; TDF, tenofovir disoproxil fumarate; VL, viral load; WHO, World Health Organization; XTC, 3TC or FTC.

^a^Viral load among viremic participants (VL ≥400 copies/mL) at switch.

### Viral Load at Switch and Follow-up

Seventy-seven participants were viremic at switch in Malawi (5.4%), compared to 42 (3.0%) in Zambia (Table 1, *P* = .001). In Zambia, the 42 participants will have become viremic since their last suppressed routine VL. Among those viremic at switch, the median VL was higher in Malawi than in Zambia: 8952 copies/mL compared to 1163 copies/mL (*P* = .017).

At 1 year, 1137 of 1422 (80.0%) participants had a VL measurement in Malawi and 1320 of 1410 (93.6%) in Zambia. The corresponding numbers at 2 years were 1140 of 1422 (80.2%) PWH in Malawi and 1248 of 1410 (88.5%) PWH in Zambia (Figure 1). At 1 year, 43 of 1137 (3.8%) PWH were viremic in Malawi compared to 25 of 1320 (1.9%) in Zambia. (Table 2, *P* = .004). At 2 years, corresponding numbers were 54 of 1140 (4.7%) and 22 of 1248 (1.8%) (Table 2, *P* < .001).

**Table 2.** Virologic Outcomes at 1 y and 2 y After Routine Switching to Dolutegravir-based First-line Antiretroviral Therapy by VL at Switch, in 2 ART Programs in Malawi and Zambia

At Switch No. of PWH (%)	At 1 Y No. of PWH (%)		At 2 Y No. of PWH (%)	
	Viremic	Suppressed	Viremic	Suppressed
Malawi N = 1422	N = 1137		N = 1140	
Viremic 77 (5.4%)	13 (22.8%)	44 (77.2%)	15 (27.8%)	39 (72.2%)
Suppressed 1345 (94.6%)	30 (2.8%)	1050 (97.2%)	39 (3.6%)	1047 (96.4%)
Relative risk (95% CI)	8.21 (4.11–15.2)		7.74 (4.18–13.3)	
*P*	<.001		<.001	
Zambia N = 1410	N = 1320		N = 1248	
Viremic 42 (3.0%)	1 (2.6%)	38 (97.4%)	2 (5.1%)	37 (94.9%)
Suppressed 1369 (97.0%)	24 (1.9%)	1257 (98.1%)	20 (1.7%)	1189 (98.3%)
Relative risk (95% CI)	1.37 (.03–7.94)		3.10 (.36–12.0)	
*P*	1.00		.30	

Relative risk (RR) of viremia (VL ≥400 copies/mL) comparing participants viremic and suppressed at switch, with exact 95% confidence intervals and *P* values. Viremia was defined as viral load ≥400 HIV-1 copies/mL and virologic suppression as VL <400 copies/mL. In Malawi, viral load was missing in 285 (20.0%) patients at 1 y and 282 (19.8%) patients at 2 y. In Zambia, VL was missing in 90 (6.4%) patients at 1 y and 162 (11.5%) patients at 2 y.

Abbreviations: ART, antiretroviral therapy; CI, confidence interval; PWH; people with HIV; VL, viral load.

In Malawi, 22.8% were viremic at 1 year among those viremic at switch (13/57), compared to 2.8% (30/1080) among those suppressed at switch (Table 2), for a relative risk (RR) of 8.21 (95% CI, 4.11–15.2). In Zambia, the corresponding percentages were 2.6% (1/39) and 1.9% (24/1281), and the RR was 1.37 (0.03–7.94). At 2 years in Malawi, 27.8% (15/54) were viremic among those viremic at switch, compared to 3.6% (39/1086) among those suppressed at switch, for an RR of 7.74 (95% CI, 4.18–13.3). In Zambia, the corresponding percentages were 5.1% (2/39) and 1.7% (20/1209), and the RR was 3.10 (0.36–12.0) (Table 2).

**Figure 2.**
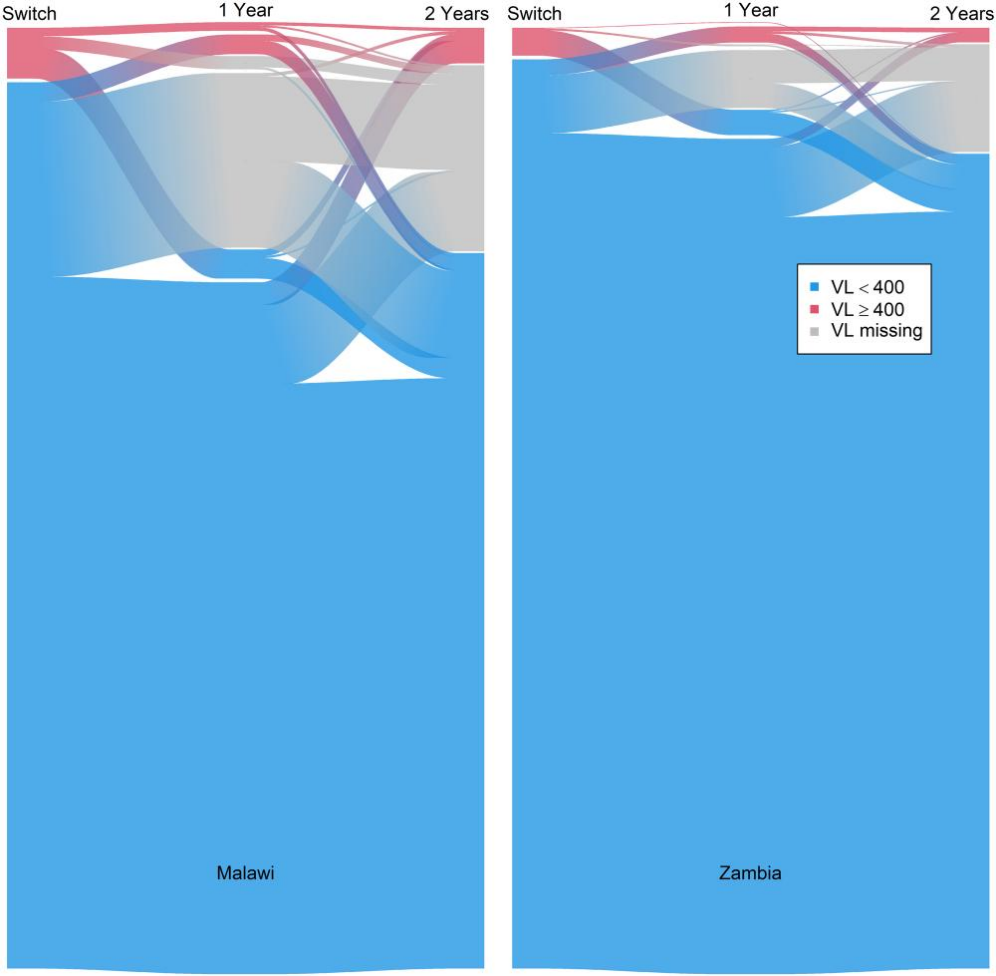
Individual trajectories of viremia (viral load [VL] ≥400 copies/mL) over the 3 time points (switch, 1-y and 2-y follow-up) in Malawi (left) and Zambia (right).

The alluvial flow diagram (Figure 2) tracks the participants’ changes between different VL states (<400 copies/mL; ≥ 400 copies/mL, missing VL) from switch to 1 year and 2 years of follow up. The diagram shows more changes between states in Malawi than in Zambia, a larger proportion with unsuppressed VL at 1 and 2 years and more missing VL values in Malawi than in Zambia. It also illustrates that only a minority (23%, 12/51) of participants who were viremic at 1 year continued being viremic at 2 years and that among those viremic at switch and suppressed at 1 year, the majority (88%, 65/77) remained suppressed at 2 years (Figure 2). Moreover, most (97%, 173/178) patients who missed their 1-year visit and returned at 2 years had suppressed VL.

### Determinants of Viremia

**Figure 3.**
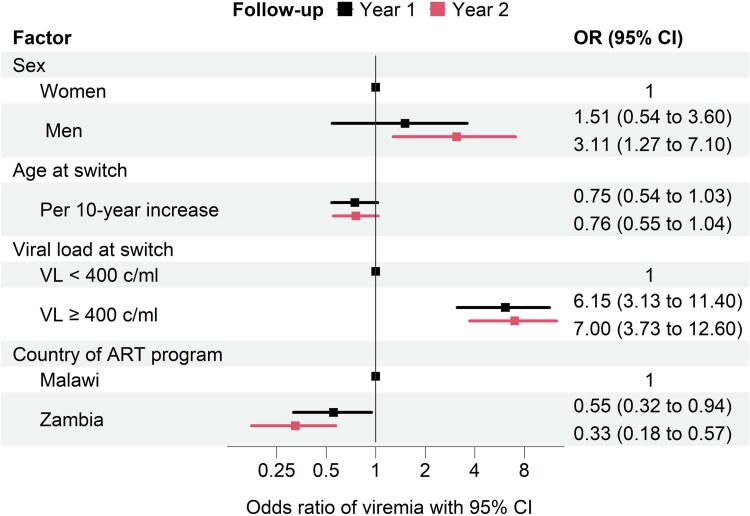
Odds ratios of viremia (viral load ≥400 copies/mL) at 1 y (first row) and 2 y (second row) after routine switching to dolutegravir (DTG)-based first-line antiretroviral therapy (ART). Results from multivariable logistic regression models adjusted for all variables shown.

The adjusted odds ratio (OR) for viremia at 1 year was 6.1 (95% CI, 3.1–11.4), comparing PWH who were viremic at switch with PWH who were virologically suppressed (Figure 3). The corresponding OR at 2 years was 7.00 (95% CI, 3.73–12.6). Men were more likely to be viremic than women, especially at 2 years (OR, 3.11; 95% CI, 1.27–7.10), and the risk of viremia declined with higher age. Finally, viremia was less likely in Zambia than in Malawi at both 1 year (OR, 0.55; 0.32–0.94) and 2 years (OR, 0.33; 0.18–0.57) (Figure 3).

The sensitivity analyses showed similar results for viremia at switch with cutoff of 1000 copies/mL (see Supplementary Table 1, Supplementary Figure 1). When modeled continuously, a 10-fold increase in baseline VL resulted in an adjusted OR of 1.92 (95% CI, 1.58–2.31) (model S2, Supplementary Figure 2). The inclusion of an interaction term between baseline VL and country (model S3, Supplementary Figure 2) indicates that the dose-response relationship was weaker in Zambia than in Malawi by factor 0.74 (95% CI, 0.43–1.15) but the interaction did not reach conventional levels of statistical significance (*P* = .22).

### Drug Resistance

There were 144 samples with VL ≥400 copies/mL at 1 year or 2 years of follow-up, from 132 study participants (92 from Malawi, 40 from Zambia). Furthermore, 113 samples contained >1000 HIV-1 copies/mL from 104 participants (75 from Malawi, 29 from Zambia). Of those, 112 samples underwent integrase sequencing, which was successful in 79 samples (70.5%) from 72 study participants (49 from Malawi, 23 from Zambia).

Drug resistance mutations in the integrase gene were observed in 5 PWH (6.9%). One participant from Malawi had major (T66A, G118R, E138K) and accessory mutations (E157Q) and 1 from Zambia major drug resistance mutations (G118R, E138K). For these participants, the Stanford interpretation system predicted high-level resistance to DTG. Three other participants from Malawi had accessory integrase mutations; they were expected to be susceptible to DTG. Two of the 5 had additional NNRTI, or NRTI and NNRTI mutations (see Table 3 for further details), in 1, the data on additional mutations were missing.

**Table 3.** List of 5 Patients With Mutations Potentially Conferring Resistance Against Integrase Strand Transfer Inhibitors (INSTI)

Country	Age at switch	HIV Subtype	Viral Load (copies/mL)			Genotypic Resistance Testing				
			At Switch	At 1 y	At 2 y	Visit	INSTI Major Mutations	INSTI Accessory Mutations	NRTI Mutations	NNRTI Mutations
Malawi	18	C	15 240	218	34 084	2 y	T66A, G118R, E138K	E157Q	D67N, K70R, M184V, K219Q	V106M, Y188L
Malawi	21	C	4891	4986	40	1 y	None	T97TA	None	K103N
Malawi	23	C	40	40	2612	2 y	None	T97A	None	None
Malawi	51	C	30	40	1260	2 y	None	A128AT	None	None
Zambia^a^	43	C	0^b^	812 791	1486	2 y	G118R, E138K	None	NA	NA

All listed participants were women on antiretroviral therapy consisting of TDF + XCT + EFV (tenofovir disoproxil fumarate plus lamivudine or emtricitabine plus efavirenz) before switching to TDF + XCT + DTG (tenofovir disoproxil fumarate plus lamivudine or emtricitabine plus dolutegravir).

Abbreviations: NA, not applicable (sequencing of reverse transcriptase was unsuccessful); NNRTI, nonnucleoside reverse transcriptase inhibitor; NRTI, nucleoside reverse transcriptase inhibitor.

^a^Sequencing was attempted at 1 y but failed.

^b^Undetectable viral load.

## DISCUSSION

The DTG SWITCH study examined virologic outcomes and emerging INSTI drug resistance in nearly 3000 PWH who were treatment-experienced but INSTI-naïve and who programmatically switched to DTG-based first-line ART in urban clinics in Malawi and Zambia. In Malawi, they switched regardless of their VL, whereas in Zambia, the national guidelines recommended only switching patients with recent VLs <1000 copies/mL. In both countries, most PWH who were viremic at switch suppressed viral replication at 1 year and at 2 years. Indeed, at all time points, the proportion of participants with suppressed viral replication was around 95% or higher, in line with the targets of the Joint United Nations Programme on HIV/AIDS to combat the global HIV/AIDS epidemic [29]. Being viremic at switch was nevertheless associated with a substantial increase in the risk of viremia later on, and 7% of participants with sequence data harbored major or accessory drug resistance mutations in the integrase gene. For 2 PWH, the Stanford algorithm predicted high-level DTG resistance.

Strengths of this study include the large sample size, the standardized protocol with a uniform follow-up of 2 years and additional viral load measurements and sequencing of the integrase gene. The study was designed to compare 2 ART programs in Southern Africa treating people with subtype C HIV infection, with different switching policies, and powered to detect differences in viremia during follow-up. Previous studies mainly enrolled people with subtype B viruses [13, 30]. The study lacked the statistical power to examine differences in the emergence of drug-resistance mutations between the 2 sites. Further, most participants were women who remained in care during the study period, and the results may not apply to men or those lost to follow-up. The national guidelines in Malawi and Zambia have recommended that adult PWH on first-line ART are switched to a DTG-based first-line regimen since 2018, except for women of childbearing age [20, 21]. In 2019, the recommendation was extended to women younger than age 45 years [2], which explains why, during the period 2019 to 2021, mostly women were switched.

Our outcome was viremia ≥400 copies/mL rather than virological failure (defined by WHO as 2 consecutive viral loads ≥1000 copies/mL [31]), although results were similar when using the higher cutoff. Viral loads were missing in up to 20% at follow-up visits in Malawi and around 10% in Zambia, which may have led to some underestimation of viremia. DTG SWITCH did not collect data on the acceptability of the new regimen or on adherence. The low prevalence of resistance mutations indicates that nonadherence to ART often explained viremia at switch and during follow-up. A study using in-depth interviews of PWH (72% women) who were programmatically switched from efavirenz-based ART to TLD in Uganda found that participants preferred the new regimen because of the smaller pill size, the convenient once-daily dosing, and the absence of side effects associated with efavirenz [32]. Sequencing was attempted in most participants who developed viremia ≥1000 copies/mL during follow-up and was successful in about 70% of them, possibly due to thawing of some samples during transport to Durban. Finally, there is evidence that mutations outside the integrase gene can confer DTG resistance [30, 33]. Our study was based on *pol* sequences, which did not allow us to investigate the effects of these mutations.

Several studies examined outcomes of PWH who were programmatically transferred to DTG-based ART in sub-Saharan Africa [14, 34–38]; 3 examined the association of viremia at switch on virologic suppression during follow-up [14, 34, 37]. A study of men and women transitioning from NNRTI-based first-line ART to DTG-based ART in rural Chiradzulu District, Malawi [34], found that 3.3% had a VL ≥1000 copies/mL at switch, compared to 4.5% in the capital of Malawi. An analysis of routine data from the East African region of IeDEA [17, 37] found that 1.1% had a VL above 1000 copies/mL preswitch, similar to our results for Zambia. The data were mainly from urban and rural Kenya and Uganda, where guidelines require a suppressed VL before transitioning to DTG-based ART [37]. The third study, from North-East Lesotho, reported that among PWH who switched first-line NNRTI-based ART to DTG-based ART, 2.2% had a VL of 1000 copies/mL or higher at switch [14]. All 3 studies found an association between viremia preswitch and viremia or virologic failure on DTG-based ART during follow-up, in line with our results.

Two previous studies examined emerging DTG resistance [14, 34]. The study from Lesotho [14] did not identify any cases of DTG resistance. The study from rural Malawi [34] found DTG resistance in 2 PWH who were viremic at switch, 1 individual with R263K and 1 with G118R mutation. In our study, both patients with major mutations had G118R and E138K, supporting previous observations that E138K occurs in combination with other mutations [30]. Systematic reviews found that in INSTI-naïve PWH, R263K and G118R are the most common mutations conferring resistance against DTG [30, 39]. We could not assess NRTI resistance at switch in our study, but the prevalence of NRTI resistance will likely have been high in viremic PWH. In rural Malawi, 60% of PWH who were viremic at switch had resistance to lamivudine, tenofovir disoproxil fumarate, or both [34]. NRTI resistance was strongly associated with DTG resistance in a collaborative analysis of cohort studies from Canada, Europe, and South Africa, and probably promotes the emergence of DTG resistance [13].

In conclusion, this large longitudinal study of treatment-experienced, INSTI-naïve PWH transitioning to DTG-based ART in 2 different settings in Malawi and Zambia highlights the infrequency of viremia postswitch. Still, it underscores the heightened risk of treatment failure in individuals with viremia at switch. The Zambian approach of switching only PWH with evidence of virologic suppression may have reduced the risk of viremia and the potential for drug resistance but the observational nature of the data preclude firm conclusions. Our findings emphasize the necessity of VL monitoring and resistance testing to maintain ART effectiveness, especially in settings with a known high prevalence of preexisting NRTI resistance. Monitoring the emergence of DTG resistance mutations is essential to prevent resistance at the individual and the population level and ensure ART's long-term sustainability.

## Supplementary Data

Supplementary materials are available at *Clinical Infectious Diseases* online. Consisting of data provided by the authors to benefit the reader, the posted materials are not copyedited and are the sole responsibility of the authors, so questions or comments should be addressed to the corresponding author.

## Supplementary Material

ciae261_Supplementary_Data

## Notes

***Author contributions.*** M. E., V. W. S., and R. L. conceptualized the study. V. W. S., M. E., and R. L. devised the methodology. V. W. S. and M. E. conducted the formal analysis and validation. V. W. S. and S. H. managed the data and provided project administration. M. E. and R. D. K. provided supervision. V. W. S. and M. E. created the figures. V. W. S. and M. E. wrote the original draft of the manuscript. All authors reviewed the manuscript. V. W. S. and M. E. directly accessed and verified the underlying data reported in the manuscript. M. E. and V. W. S. had full access to all the data in the study. All authors had the final responsibility for the decision to submit for publication.

***Acknowledgments.*** The authors are grateful to all the patients participating in this study, the study nurses, the data clerks, and data managers. Special thanks to Alice Miyanda, Kenan Simumba, Caroline Chileshe, Mary Chipula, Nyanyiwe Tembo, Olpha Malora, Jane Nkhono, and Rafique Maluwa for patient recruitment and fieldwork, and to Lavanya Singh and Nonkululeko Avril Magini for sequencing work.

***Data sharing.*** Deidentified participant data and a data dictionary can be made available and shared under a data transfer agreement. Requests for access to data should be sent to matthias.egger@unibe.ch. Nucleotide sequences will be made available on GenBank pending approval by local authorities.

***Financial support.*** This study was supported by the National Institutes of Health (NIH) National Institute of Allergy and Infectious Diseases under award number R01AI152772 and the Swiss National Science Foundation (32FP30_207285, 324730_207957). Centre for Infectious Disease Research in Zambia (CIDRZ) and Lighthouse Trust are supported via International epidemiology Databases to Evaluate AIDS in Southern Africa (IeDEA-SA) by the following NIH institutes: the National Institute of Allergy and Infectious Diseases, the Eunice Kennedy Shriver National Institute of Child Health and Human Development, the Division of Cancer Epidemiology and Genetics of the National Cancer Institute, the National Institute of Mental Health, the National Institute on Drug Abuse, the National Heart, Lung, and Blood Institute, the National Institute on Alcohol Abuse and Alcoholism, the National Institute of Diabetes and Digestive and Kidney Diseases, and the Fogarty International Center under award number U01AI069924.
