# Supplementary material for: Virologic Failure and Drug Resistance After Programmatic Switching to Dolutegravir-based First-line Antiretroviral Therapy in Malawi and Zambia
Source: Clin Infect Dis. 2024 Jun 7;80(1):120–8. doi: 10.1093/cid/ciae261 (PMC11797054; doi:10.1093/cid/ciae261)
Supplement: ciae261_Supplementary_Data [file ciae261_supplementary_data.pdf]

Virologic failure and drug resistance after programmatic switching to dolutegravir-based first-line ART in Malawi and Zambia

Supplementary materials

Supplementary Table 1. Virologic outcomes of PWH at 1 year and 2 years after routine switching to DTG-based first-line ART by viral load at switch, and relative risk (RR) of viremia with exact 95% confidence intervals (CI) and P-values, in two ART programs in Malawi and Zambia. Sensitivity analysis using cut-off 1000 copies/mL.

		Viral load* (No. of patients)			
**At switch**	No. of PWH (%)	**At 1 year of follow-up** No. of PWH (%)		**At 2 years of follow-up** No. of PWH (%)	
		Viremic	Suppressed	Viremic	Suppressed
**Malawi** N=1422		N=1137		N=1140	
Viremic	64 (4.5%)	11 (19.3%)	46 (80.7%)	14 (25.9%)	40 (74.1%)
Suppressed	1358 (95.5%)	25 (2.3%)	1055 (97.7%)	29 (2.7%)	1057 (97.3%)
Relative risk (95% CI)		8.34 (3.85 to 16.5)		9.71 (4.98 to 17.7)	
P		<0.001		<0.001	
**Zambia** N=1410		N=1320		N=1248	
Viremic	26 (1.8%)	1 (2.6%)	38 (97.4%)	1 (2.6%)	38 (97.4%)
Suppressed	1384 (98.2%)	15 (1.2%)	1266 (98.8%)	17 (1.4%)	1192 (98.3%)
RR (95% CI)		2.19 (0.05 to 13.5)		1.82 (0.04 to 11.0)	
P		0.77		0.88	

* Viremia was defined as viral load (VL) ≥1000 copies/mL and virologic suppression as VL <1000 copies/mL.
Viremia was defined as viral load ≥400 HIV-1 copies/mL and virologic suppression as VL <400 copies/mL.
In Malawi viral load was missing in 285 (20.0%) patients at 1 year, and 282 (19.8%) patients at 2 years.
In Zambia, viral load was missing in 90 (6.4%) patients at 1 year and 162 (11.5%) patients at 2 years.
PWH; people living with HIV

Supplementary Figure 1. Odds ratios of viremia (viral load ≥1000 copies/mL) at 1 year (black) and 2 years (magenta) after routine switching to DTG-based first-line ART, with 95% confidence intervals (CI). Results from logistic regression models adjusted for all variables shown (Model S1).

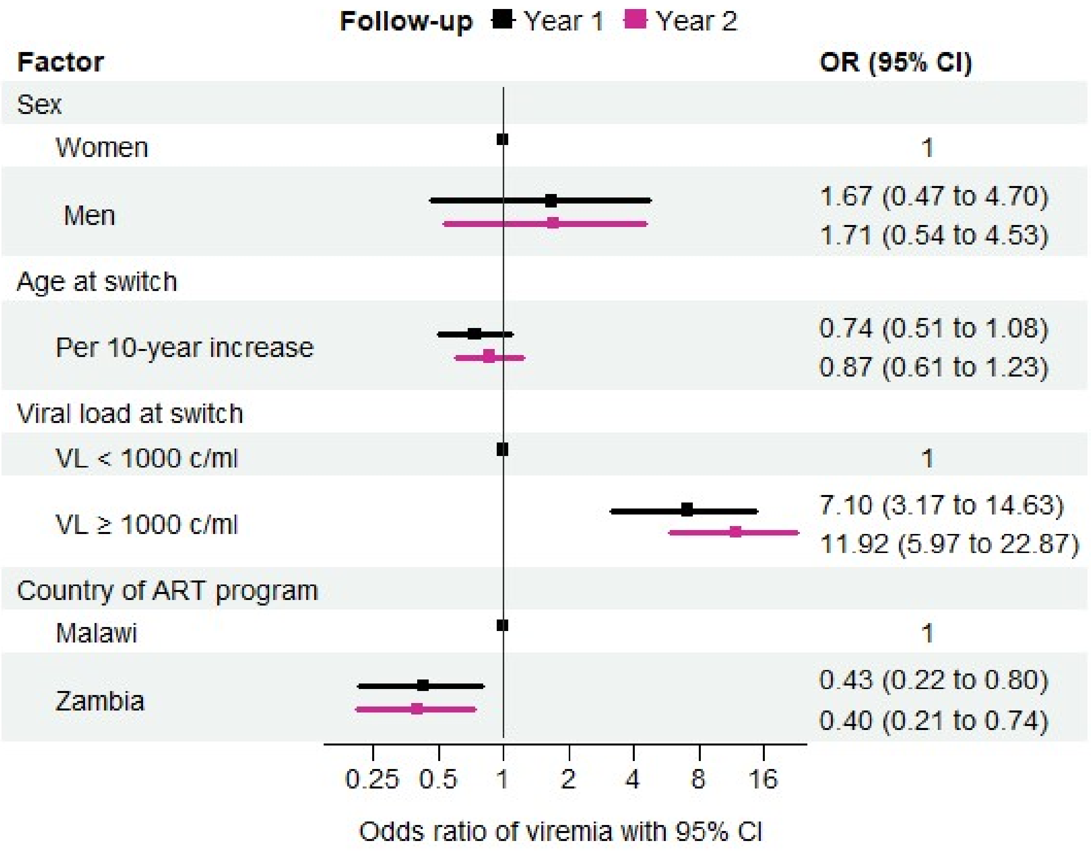

Supplementary Figure 2: Odds ratios of viremia (viral load ≥400 copies/mL) at 2 years after routine switching to DTG-based first-line ART, with 95% confidence intervals (CI), based on model without interaction (black) and with interaction (blue) (Models S2 and S3). Viral load (VL) at switch was modelled continuously as log10-transformed VL with a set-off 10 for undetectable VL measurements.

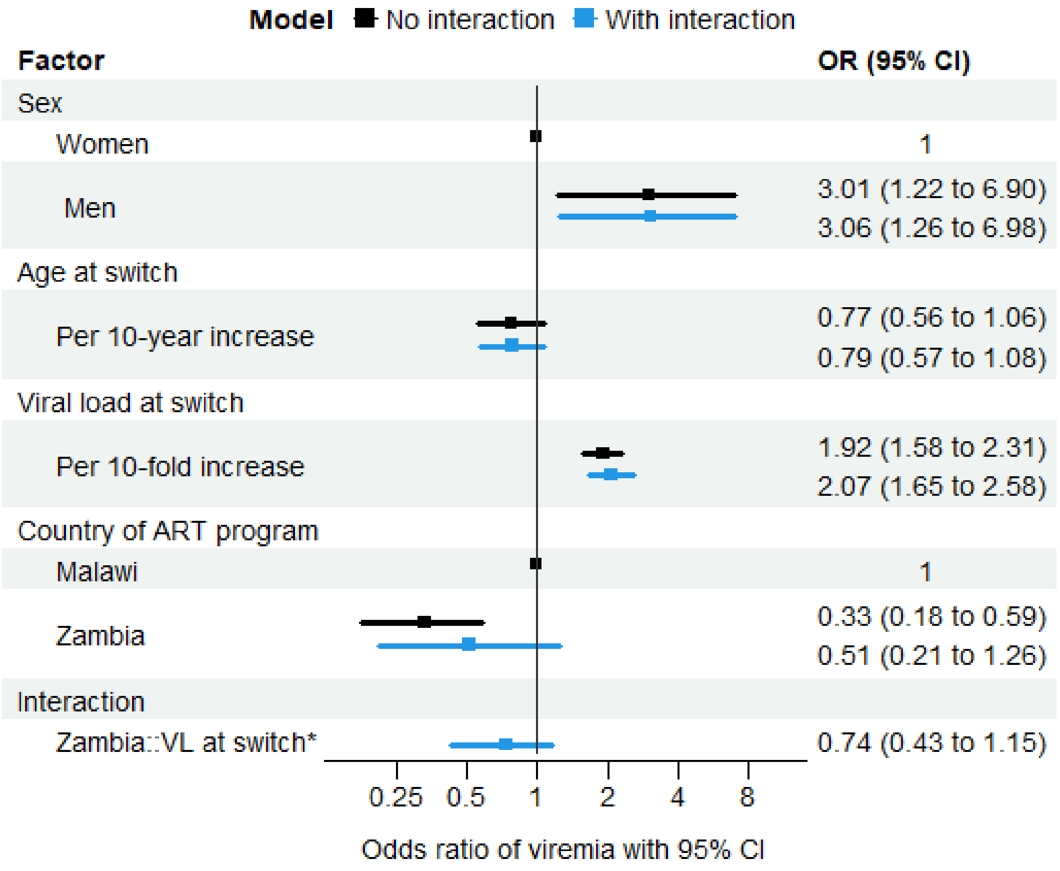

*The interaction corresponds to the contrast in OR (per 10-fold increase in VL at switch) between Zambia and Malawi.
